# Celiac Disease and the Microbiome

**DOI:** 10.3390/nu11102403

**Published:** 2019-10-08

**Authors:** Francesco Valitutti, Salvatore Cucchiara, Alessio Fasano

**Affiliations:** 1 Pediatric Gastroenterology and Liver Unit, Department of “Maternal-and-Child Health” and Urology, Sapienza University of Rome, 00185 Rome, Italy; salvatore.cucchiara@uniroma1.it; 2 European Biomedical Research Institute of Salerno, 84125 Salerno, Italy; AFASANO@mgh.harvard.edu; 3 Center for Celiac Research and Treatment, Mucosal Immunology and Biology Research Center, MassGeneral Hospital for Children, Boston, MA 02114, USA; 4 Division of Pediatric Gastroenterology and Nutrition, MassGeneral Hospital for Children, Boston, MA 02114, USA

**Keywords:** celiac disease, microbiome, microbiota, environmental factors, at-risk infants

## Abstract

Growing evidence supports the hypothesis that changes in both the composition and function of the intestinal microbiome are associated with a number of chronic inflammatory diseases including celiac disease (CD). One of the major advances in the field of microbiome studies over the last few decades has been the development of culture-independent approaches to identify and quantify the components of the human microbiota. The study of nucleic acids DNA and RNA found in feces or other biological samples bypasses the need for tissue cultures and also allows the characterization of non-cultivable microbes. Current evidence on the composition of the intestinal microbiome and its role as a causative trigger for CD is highly heterogeneous and sometimes contradictory. This review is aimed at summarizing both pre-clinical (basic science data) and clinical (cross-sectional and prospective studies) evidence addressing the relationship between the intestinal microbiome and CD.

## 1. Introduction

Although the recognition of the causal link between gluten and celiac disease (CD) was unveiled in the 1950s [1], what factor (or factors) triggers the loss of immune tolerance to gluten in genetically predisposed subjects still remains unknown. Since its original description, CD has most often been perceived as a pediatric condition with a peak incidence in children younger than two years of age, with more recent data suggesting that most of the cases would manifest by five years of age [2].

The worldwide prevalence of CD ranges between 1% and 2% in the general population [3,4], with most patients remaining undiagnosed due to the subtle or multiform clinical manifestations of the disease [5]. Based on more recent epidemiological data, and contrary to the original paradigm, it is now appreciated that CD can present at any age with a broad range of intestinal and extra-intestinal symptoms [6,7]. Its prevalence, as in many other autoimmune diseases often found in comorbidity with CD [8], has increased over time in geographical regions characterized by a Western lifestyle [9]. This phenomenon was initially hypothesized to be secondary to the timing of gluten introduction at weaning [10], although two large, randomized, and prospective high-risk, birth cohort-controlled trials have disputed this premise by demonstrating that neither delayed nor early gluten introduction modified the risk of CD [2,11].

These findings raised doubts about another CD paradigm that suggested that genetic background and dietary gluten intake were necessary and sufficient to develop the disease. Besides the evidence that CD onset can occur years after gluten introduction into the diet [6], other evidence at odds with the old paradigm is the lack of 100% CD concordance among monozygotic twins [12]. Therefore, while genetic predisposition (including the required presence of HLA DQ2 and/or DQ8 haplotypes) and gluten exposure are necessary, they seem to be insufficient for the development of CD autoimmunity. Intestinal permeability is an additional element involved in CD pathogenesis, as a “leaky gut” might initiate the early phases of innate immune activation following the exaggerated trafficking of undigested gluten fragments from the intestinal lumen to the lamina propria [13].

Growing evidence supports the hypothesis that changes in gut microbiome composition and function are associated with a number of chronic inflammatory diseases including obesity [14], diabetes [15], inflammatory bowel disease [16] and cancer [17]. This might also be the case for CD.

In the last decades, one of the major advances in the field of microbiome studies has been the ability to apply culture-independent approaches to determine the microbiome’s composition [18]. These technologies allow for the identification and quantification of components of the human microbiota by studying nucleic acids (DNA and RNA) from fecal samples or other biological samples [19], which eliminates the need for tissue cultures and also allows the characterization of non-cultivable microbes.

The human gastrointestinal lumen contains a copious and diverse microbial ecosystem of over 100 trillion microorganisms [20]. More than 2 million genes are expressed by the human microbiome, and these genes encode for metabolic pathways that finally produce thousands of metabolites [21]. Conversely, it is striking to note that the human genome is composed of only 23,000 genes [22]. Consequently, the host and its microbial communities can be viewed as a “superorganism” with mutable immune and metabolic profiles [23].

Gut bacteria facilitate the digestion of insoluble fiber, produce vitamins such as vitamin K, and elaborate trophic and immunomodulating compounds such as short-chain fatty acids (SCFA) [24].

Moreover, they also display key immune-modulating functions within the gut. By competing for nutritional sources and producing anti-microbial molecules, beneficial gut bacteria counterbalance the growth of pathogenic bacteria and favor epithelial integrity [25,26]. Microbiome-derived SCFA can also modulate host histone deacetylase, therefore epigenetically influencing the function of innate and adaptive immune cells [27]. The impact of the gut microbiome on mucosal immunity is further demonstrated by the evidence of defects in lymphoid tissues (a decreased number of mucosal Peyer’s patches and smaller mesenteric lymph nodes) and compromised antibody production in germ-free animals [28].

In early childhood, microbial diversity rises with age until it stabilizes with two major bacterial phyla: *Firmicutes* and *Bacteroidetes*, which represent roughly 90% of the whole gut microbiota. *Actinobacteria, Proteobacteria, Fusobacteria* and *Verrucomicrobia* are the next most-numerous describing a “healthy gut microbiota composition” [29]. At approximately three years of age, a child’s gut microbiota composition and diversity are very similar to the adult microbiota [30]. While it is generally assumed that microbiome engraftment occurs at birth during the passage through the vaginal canal, or via maternal skin microbiota in case of cesarean section, there are a few reports showing that a specific microbiota colonizes the placenta [31] and is detectable in the meconium [32], suggesting that engraftment may start in utero.

In recent years, research into the early development of the microbiome has highlighted the influences of delivery mode, maternal/infant nutrition and antibiotics on the engraftment and subsequent changes in intestinal microbiome composition [33,34]. This crucial initial symbiotic relationship between host and gut microbiome is instrumental in programming the immune system to distinguish between pathogens and commensals to achieve the proper strategies to unleash inflammation when necessary (for example fighting pathogens) or maintain anergy [35].

This review is aimed at summarizing current evidence on the relationship between the gut microbiome and CD. For the sake of brevity, no studies on the microbiomes of patients on a gluten-free diet (GFD) have been considered. This choice is also justified by the fact that gluten dietary exclusion would represent an “intervention” affecting gut microbiome composition, thus introducing a strong bias for further considerations.

The literature search was run using Pubmed, EMBASE, Web of Science and Scopus using terms as: ‘‘microbiome and CD’’ (341 articles), ‘‘microbiota and CD” (301 articles), “gut microbiome and CD” (152 articles) and ‘‘gut microbiota and CD” (220 articles). The search was limited to articles written in English. All abstract papers were read, 153 were analyzed as full articles, and finally, only 129 were included as references for this review.

## 2. Microbiome, Environmental Factors and Gut Inflammation: Implications for Celiac Disease (CD)

Environmental factors strongly drive microbiota engraftment and subsequent composition. For example, vaginal delivery ensures the vertical mother–infant transmission for pivotal gut microbiome components such as *Bacteroides* and *Bifidobacteria* [36]. Conversely, cesarean (C) section-born infants show less *Bacteroidetes*, and the diversity of this specific phylum is lower [37]. However, while it is uncertain if these changes might explain some reports of an increased risk of CD for children born via C-section [38,39], it should be acknowledged that the association between C-section and CD is still controversial [40].

Diet is another key regulator of microbiome development and homeostasis. The human milk oligosaccharides (HMOs) select the growth of commensals such as *Bifidobacteria* and prevent the growth of potential pathogens such as *Clostridium difficile* [41,42]. Moreover, HMOs enhance overall barrier integrity by making enterocytes less vulnerable to bacterial-induced innate immunity [43]. Therefore, breast-feeding seems to be ideal for the engraftment of a symbiotic gut microbiome.

Some data also suggested that maternal antibiotic assumption during pregnancy shapes the gut microbiota in the offspring [44], albeit a cohort study found no statistically significant association between maternal use of antibiotics during pregnancy and CD risk in the offspring [45]. According to some reports, antibiotic exposure during the first year of life has been associated with an increased risk of developing CD [46,47], however, other studies did not confirm this finding [48,49,50]. A recent meta-analysis did not resolve these incongruences, albeit favoring a non-causal relationship between early antibiotics exposure and CD [51].

Early life infections may be involved in CD onset, and this issue is also supported by cohort studies [52,53]. Another study that looked at the effect of viral triggers and Th1 response recognized reovirus as a possible cofactor for both inappropriate immune activation and subsequent loss of tolerance to gliadin [54]. Patients with CD display higher antibody titers against human adenovirus serotype 2 [55,56]. This might go along with the clinical interpretation of in vivo data. A longitudinal prospective cohort of genetically at-risk children demonstrated that an increased rate of rotavirus gastroenteritis may strengthen the risk of CD in infancy [57]. However, the implementation of rotavirus vaccination did not prevent a rise in CD prevalence that has been recently reported in Italian children [58]. A role for *Candida albicans* in CD development has been hypothesized based on sequence similarities between a hyphal wall protein and several T-cell gliadin epitopes [59], albeit the only small study on mycobiome next-generation sequencing analysis of duodenal samples showed no difference between adult CD cases and controls [60]. A large cohort study from Sweden has shown that there is a significantly higher hazard ratio of *C. difficile* infection in patients with CD when compared to age- and gender-matched controls [61], albeit study limitations leave open a few areas of uncertainties [62].

The physical isolation of microbes from the glycocalyx of the intestinal epithelium without evidence of overt inflammation suggests that preventing physical contact with the gut mucosa avoids activation of the immune system, therefore favoring a symbiotic relationship between the host and the gut microbiome [63]. A balanced gut microbiota also contributes to the maintenance of the mucous layer, especially due to bacteria such as *Lactobacillus* species and *Akkermansia muciniphila* [64,65]. A healthy microbiota additionally favors colonization resistance, namely, the capability of commensal bacteria to compete for nutrients with pathogens, thereby stimulating the epithelium to secrete antimicrobial molecules into the mucous layer and provide a better defense against pathogens [66]. Additionally, commensals contribute to this line of defense by synthesizing protective substances, such as acetate produced by *Bifidobacterium*, which prevents colonization by *enterohemorrhagic E. coli* O157:H7 [67]. IgAs produced by the gut-associated lymphoid tissue (GALT) also contribute to barrier maintenance, microbiome selection and decreased activation of innate immunity [68]. Related to this topic, Olivares et al. demonstrated that a reduction in IgA fecal level can precede CD development in infants [69].

Some studies have shown an increased expression of genes responsible for pathogen-associated molecular pattern (PAMPs) recognition, such as Toll-like receptors (TLR) in CD. For example, Szebeni et al. found higher expressions of TLR2 and TLR4 in untreated and treated CD patients versus controls, as shown both at mRNA and protein levels [70]. Furthermore, TLR2, TLR9 and TOLLIP, an intracellular protein that inhibits TLR, have been found as microbiota-associated factors in the possible development of CD [71]. Overexpression of TOLLIP in vitro offsets TLR pathways after lipopolysaccharide or lipotechoic acid stimulation. This phenomenon has been named “lipopolysaccharide tolerance” [72]. In fact, a reduced expression of TOLLIP in active CD might indicate that a failure to tolerate microbiota may contribute to CD immune activation.

It is well acknowledged that the host can tightly control the microbiota, however, the microbiota also exerts a strong programming on host metabolism and immunity [73]. SCFA synthesized by commensal bacteria condition regulatory T-cells (Treg cells), specifically, one member of the SCFA, butyrate, helps T-cells to differentiate toward Treg cells [74]. SCFA might inhibit histone deacetylases, provoking hyperacetylation of histones, which finally results in anti-inflammatory gene activation [75].

The role of gut microbiota and their metabolites in CD has been explored by a recent study showing their effects on Treg cells through epigenetic processes [76]. Specifically, CD patients showed an increased expression of a non-functional spliced form of FOXP3 (so increasing the risk of developing autoimmunity) which could be attributable to the altered intestinal microbiota and to its unbalanced butyrate production.

In another study, CD-derived organoids treated for 48 h with microbiota-derived compounds, such as lactate, butyrate and polysaccharide A, showed a significant improvement of intestinal permeability measured as transepithelial electrical resistance changes. Moreover, the same group also showed that butyrate significantly upregulated the expression of genes regulating epithelial integrity in CD organoids [77].

It has been noticed that the HLA-DQ genotype can affect early gut microbiota composition [78], and an increased occurrence of pathogenic bacteria such as *enterotoxigenic Escherichia coli* has also been described in infants genetically at risk for CD [79]. In a previous study from a Spanish group, higher numbers of *Bifidobacterium* spp. and *Bifidobacterium longum* were present in the gut microbiota of infants with the lowest HLA-DQ genetic risk for CD, whereas, for those with the highest genetic risk, higher *Staphylococcus* spp. and *Bacteroides fragilis* were identified. However, the method of infant-feeding influenced the composition of the microbiota, with breast milk favoring *Clostridium leptum*, *Bifidobacterium longum* and *Bifidobacterium breve* gut colonization, therefore slightly switching the fecal microbiome toward the one identified in infants with low HLA-DQ genetic risk [80].

## 3. Bifidobacteria and Lactobacilli Strains: Few “Paladins” in the Pathogenetic Joust?

In the pursuit of the best microbial candidate for disease immunomodulation, a few *Bifidobacteria* strains have been studied with considerable results. For example, in an in vitro model using peripheral blood mononuclear cell (PBMCs), both *Bifidobacterium longum ES1* and *Bifidobacterium bifidum ES2* have been shown to downregulate the Th1 pathway typical of CD [81].

In addition, Lindfors et al. assessed whether *Bifidobacterium lactis* is capable of neutralizing the toxicity of gliadin. In Caco-2 cells, they found that this strain was at least able to reduce the epithelial permeability triggered by gluten [82].

Laparra et al. evaluated *Bifidobacterium longum* CECT 7347 in a murine model of CD, and they found that this specific strain not only diminishes pro-inflammatory cytokine synthesis, such as tumor necrosis factor-alfa (TNF-alfa), but it also reduces jejunal architecture damage [83]. Another group has demonstrated that *Bifidobacterium longum* strain NCC2705 produces a serine protease inhibitor with immune-modulating features, i.e., attenuating gliadin-induced histological damage in NOD/DQ8 mice [84].

An alternative *Bifidobacterium, B. infantis*, seems to decrease Paneth cells and expression of alfa-defensin-5 on electronic microscopy of duodenal biopsy when administered in active CD [85]. Paneth cells are key masters of gut homeostasis in innate immunity against noxious pathogens through the release of defensins, lysozyme and phospholipase [86]. Furthermore, some evidence concerning the protective effect of *Lactobacillus casei* DN-114001 and *E. Coli strain Nissen* 1917 on gut barrier function has been reported [87].

D’Arienzo et al. analyzed the effect of *Lactobacillus casei, Lactobacillus paracasei* and *Lactobacillus fermentum* in a transgenic mouse model expressing human DQ8. They found that *L casei* reduces TNF-alfa secretion and related villous blunting, while both *L paracasei* and *L fermentum* determine increased antigen-specific TNF-alfa. This suggests that, depending on the strain and on the experimental model, probiotics may have either proinflammatory or immunomodulatory properties [88,89].

## 4. Focus on Other Species and Strains: The Joust Gets Hectic

Several other bacterial species and specific strains have been studied in regard to their possible link with CD pathogenesis. As *Bacteroides fragilis* clones expressing metalloproteases were often reported in patients with CD, this might underscore an anticipated role played in CD pathogenesis. *B. fragilis* strains carrying metalloprotease genes may lead to increased intestinal permeability and production of gliadin immunogenic peptides. Furthermore, these peptides could maintain or even intensify their ability to provoke an inflammatory response mediated by TNF-alfa. These increases in TNF-alfa production by epithelial cells could have deleterious effects that fuel both innate and adaptive immunity in CD onset [90].

Some *Prevotella* species, *Lachnoanaerobaculum umeaense* and *Actinomyces Graevenitzii*, were isolated from CD jejunal biopsies. These species could trigger an IL-17A-driven immune response [91]. This emphasizes the possibility that the increased IL-17A response seen in active CD could be in part attributable to host-microbiota interactions, and this might also explain why the IL-17A mucosal response in CD is not consistent in some CD patients [92].

*Neisseria flavescens* determines inflammation and induces disturbances in the mitochondrial chain processes of Caco-2 epithelial cells. This latter metabolic alteration seems to be partly corrected when *Lactobacillus paracasei* CBA is administered [93]. Another study involving *N. flavescens* showed that five different strains isolated from adults with untreated CD led to an inflammatory activation of both human and murine dendritic cells (DC) [94]. Nevertheless, it is not clear whether *N. flavescens* causes inflammation, or the inflammatory process occurring in the gut of CD patients may favor its colonization, which then simply maintains an activated proinflammatory response.

In addition, it has been demonstrated by Galipeau et al. that gut microbiota can either reduce or exacerbate gliadin-induced damage in a mouse model of CD [95]. In this study, the expansion of the Proteobacteria phylum caused more severe intestinal damage induced by gluten. This could possibly be explained by the fact that the intestinal mucus layer is more penetrable to bacteria and toxins where Proteobacteria prevail [96]. Similar evidence comes from a study on Caco-2 cells from Spain. *Enterobacteriaceae* (belonging to the Proteobacteria phylum) were found to act similarly to gliadins regarding DC maturation, i.e., attachment, spreading and pro-inflammatory cytokine polarization. On the other hand, *Bifidobacterium longum* CECT 7347 counterbalanced IFN-production as a consequence of gliadin stimulation and increased IL-10 release [97]. Altogether, these evidences underline the importance of the biological milieu of the intestinal lumen for disease advancement.

## 5. Microbiome- Derived Gluten-Degrading Enzymes: Opportunity for Prevention and Alternative Treatment

Another issue to consider is the capacity of the enzyme machinery belonging to the gut microbiome to completely digest gluten. To this extent, it is of note that, after the bacterial proteolytic degradation of gliadin, peptides could still be toxic and eventually cross the intestinal barrier more easily [98]. However, few in vitro studies have revealed that microbiota components, specifically *Bifidobacteria*, can degrade proinflammatory gluten peptides in the small intestine, thus reducing their immunogenic potential [82,99]. In one recent study, some *Lactobacilli* were able to digest in vitro amylase-trypsin inhibitors (ATIs), non-gluten wheat proteins that induce an innate immune response through the Toll-like receptor 4 (TLR4)–MD2–CD14 mechanisms. It is of note that the administration of *Lactobacilli* species (*Lactobacillus salivarius H32.1*, *Lactobacillus mucosae D5a1* and *Lactobacillus rhamnosus LE3*) decreased both inflammation and permeability stimulated by ATIs [100].

Along with the bacterial component of the gut microbiome, enzymes able to digest gluten can also be elaborated by some eukaryotes. Papista et al. studied the influence of *Saccharomyces boulardii* KK1 supplementation in an animal model of gluten enteropathy (BALB/c mice). This intervention allowed the hydrolyzation of toxic gliadin peptides and counterbalanced both enteropathy and pro-inflammatory cytokine production [101]. In line with these data, another group has shown degrading activities toward toxic gluten epitopes by oral commensal bacteria such as *Rothia* spp, *Actinomyces odontolyticus, Neisseria mucosa* and *Capnocytophaga sputigena* [102]. Currently, some drugs based on degrading enzymes from bacteria and fungi have been used in clinical trials with diverse results [103].

It is well established that compliance to the GFD is difficult [104], and, for this reason, there is a great expectation among CD patients for drug-based therapies [105]. In light of these challenges, these findings on gluten-degrading activities by specific microbial strains might pave the way for a probiotics-based complementary therapy of CD in the years to come.

## 6. Cross-Sectional Studies on CD and the Microbiome

Studies of electron microscopy scans of the small intestine have shown that some bacteria were most frequently detectable in young patients with CD during the so-called Swedish epidemics [106,107]. These rod-shaped bacteria adhering to the epithelial lining in the small intestine were commonly seen in children with CD but not in controls.

In 2007, a Spanish study showed that the diversity of stool microbiota was significantly higher in CD children than in healthy controls, and that *Bifidobacteria* showed a significantly higher species diversity in healthy children than in CD [108]. In the same year, the same group found (using small bowel biopsy samples) that the proportions of total bacteria and Gram-negative bacteria were significantly higher in CD children with active disease than in controls. *Lactobacillus–Bifidobacterium* were significantly reduced while *Bacteroides–E. coli* were significantly increased in active CD compared with controls [109]. Collado et al. showed that *Clostridium leptum, Bacteroides, Staphylococcus* and *E coli* were significantly more abundant in stool and biopsy samples of pediatric CD patients than in healthy controls. Conversely, *Bifidobacterium* appeared significantly lower in feces of CD children as well as in biopsies compared to control children [110].

Another study on fecal samples also showed that *Bacteroides* and *Prevotella* were higher in untreated pediatric CD than in controls, whereas *Clostridium histolyticum, Clostridium lituseburense* and *Faecalibacterium prausnitzii* were higher in healthy individuals than in CD children. According to previous data, *Bifidobacteria* as well were significantly reduced in untreated CD [111]. Active CD patients were reported to have a higher abundance of *Bacteroides fragilis* and a lower abundance of *Bacteroides ovatus* than controls [90].

In 2010, a study from Italy pinpointed that a higher diversity in dominant microbiota characterized CD children compared to controls. In addition, *Bacteroides* were significantly higher in CD compared to controls [112]. A subsequent study from Spain demonstrated that *Proteobacteria* were more abundant in duodenal biopsies of active CD children than in those of controls [113]. Nevertheless, two different studies from Finland, one study from the Netherlands and one more study from Spain did not replicate previous findings, revealing no significant differences in small bowel biopsies concerning the amounts or frequencies of bacteria identified between the CD subjects and controls [71,114,115,116].

In adult subjects, researchers from Spain found that bacterial richness in the upper small intestinal mucosa is higher in adults than in children with CD [117]. However, in another work published in the same year, the authors demonstrated that *Bifidobacterium bifidum* was significantly higher in the stool samples of untreated CD patients than in those of healthy adults [118]. In another study, *Helicobacter* and *Megasphaera genera* were highly abundant in duodenal biopsy samples from adult CD patients compared to both first-degree relatives and the control group. Conversely, *Barnesiella* were higher in controls compared to CD and first-degree relatives in duodenal samples, while *Dorea, Akkermansia* and *Prevotella* genera were higher in fecal samples from controls compared to CD [119]. In 2016, D’Argenio et al. demonstrated that *Proteobacteria* were more abundant in samples from duodenal biopsies of adult subjects, while *Firmicutes* and *Actinobacteria* were not as well represented in active CD compared to controls. In the *Neisseriales* order, the *Neisseriaceae* family and the *Neisseria genus* were significantly more present in active CD patients than in controls [94].

However, a word of caution concerning the studies mentioned above must be noted. Studies focused on duodenal and/or jejunal microbiome in CD are scarce and often present contrasting results. Additionally, differences in mucosal microbiome composition may represent the consequence of an inflamed CD mucosa, rather than a contribution to CD pathogenesis. Ideally, comparable intestinal and fecal microbiome analyses in the same subject may shed light on possible differences to be considered in interpreting these data.

Adult patients with *dermatitis herpetiformis* seem to share more similar microbiota with control subjects than those with other clinical features of CD. In fact, patients with gastrointestinal symptoms had a higher amount of *Proteobacteria* than patients with another manifestation of the disease (i.e., *dermatitis herpetiformis*) and the control subjects [120]. This finding allows speculation into the possibility that microbiota might drive the symptoms of CD, which might also explain its protean clinical features.

In regard to specific pathogenic features of bacterial strains associated with CD, studies from Spain highlighted that virulence-gene carriage was higher in *E. coli,* in samples isolated from the stool of children with CD when compared to healthy controls [121], and that the methicillin-resistant gene (mecA) was most frequently identified in *Staphylococcus epidermidis* isolated from stools of active CD than in those from controls [122]. All cross-sectional studies are recapitulated in Table 1.

To summarize, considering all these cross-sectional studies in a comprehensive fashion, it should be pointed out that the highly individual-specific microbial profiles may greatly impact the interpretation of the results, especially when evaluated on relatively small groups (a common feature of many studies published to date). Moreover, another limitation of these results is the spurious healthy control group, especially in regard to patients who underwent an upper GI endoscopy because of signs or symptoms of disease, the microbiomes of these individuals might have hosted unpredictable microbiota alterations. In addition, a strong limitation does apply for those studies that utilized PCR-D/TGGE analysis (see Table 1), solely detecting the most-represented bacteria and therefore underestimating microbiota diversity. Finally, and specifically for the mucosa-associated microbiome, it is unclear whether changes in microbiota strains colonizing the duodenal mucosa influenced by environmental risk factors (i.e., infant-feeding practice, exposure to antibiotics and use of anti-acids, etc.) could be the cause of CD, or that structural and histological changes characterizing the celiac enteropathy are responsible for secondary shifts in the composition of the adherent microbiota. Despite all these limitations, one might conclude that a decrease in *Bifidobacteria* and an increase in *Bacteroides* seem to be a somewhat common denominator of a few studies, both on feces and on mucosal biopsies.

## 7. Prospective Cohort Studies on Microbiome in CD

The only chance to detect a contributory microbial signature of CD and to mechanistically correlate potential environmental factors involved in disease onset is to follow-up cohorts of infants at risk for CD in a prospective manner. It has been previously reported that HLA genotype per se affects the gut microbiota of infants at family risk for CD, with DQ2-positive subjects displaying higher abundance of *Firmicutes* and *Proteobacteria* and lower abundance of *Actinobacteria* [123]. Infants with high genetic risk for CD showed a higher prevalence over time of *Bacteroides vulgatus*, while those with low genetic risk displayed a higher prevalence of *Bacteroides ovatus, Bacteroides plebeius* and *Bacteroides uniformis* [124].

Sellitto et al. analyzed stool samples from a relatively small number of subjects at several time-points (7 days and 30 days, 6 months, 8 months, 10 months, 12 months, 18 months and 24 months). Their data suggested significant differences between the developing microbiota of infants with a genetic predisposition for CD compared to those from infants with a non-selected genetic background. In their proof-of-concept study, they recruited infants with a genetic predisposition for CD and assessed them prospectively until 24 months of age. 16s gene analysis proved that compared with the low-risk subjects, infants carrying the CD-associated HLA had increased *Firmicutes* and *Proteobacteria*, while *Actinobacteria* and *Bacteroidetes* were significantly restricted. Additionally, they also found that stool microbiota in these DQ2+/DQ8+ children did not stabilize, nor was it similar to adult microbiota at one year of age, and this feature remained at 24 months of age. In the only infant who developed CD during the follow-up, Sellitto et al. found a lactate peak between 6 and 12 months of age, speculating about a possible microbiome disturbance at that time [125].

Two more studies have been published from the PROFICEL prospective cohort in Spain by the same group. When researchers examined stool samples from infants at genetic risk for CD at 4 and 6 months of age, those who did not develop CD showed an increased bacterial diversity over time. Furthermore, a higher abundance of *Bifidobacterium longum* was found in control children, while higher levels of *Bifidobacterium breve* and *Enterococcus* spp. were found in those who developed CD [68]. When researchers examined stool samples from infants at genetic risk within the first week of life, and at 4 months and at 6 months of age, *enterotoxigenic E. coli (ETEC)* were found more often in infants with the highest genetic risk versus those with a low or intermediate risk among breastfed infants. Among infants on formula feeding, on the other hand, a higher number of ETEC was also identified in infants with a high genetic risk versus those of intermediate risk [79]. Albeit limited to fewer time-point assessments (4 months and 6 months), another prospective study from Finland did not find any significant difference in fecal microbiota composition between children who later developed CD and control children without disease or associated autoantibodies [126]. All prospective studies are summarized in Table 2.

## 8. Conclusions

Current evidence into the composition of the intestinal microbiome and its role as a causative trigger for CD is highly heterogeneous and contradictory. This is most frequently due to the limited number of cross-sectional and prospective studies performed, small sample sizes and different methodologies applied (fluorescence in situ hybridization-PCR, denaturing gradient gel electrophoresis and 16s ribosomal RNA sequencing). In the recent past, molecular microbiology has developed based on the analysis of bacterial DNA, therefore bypassing the shortfall of microbiome analysis limited to its cultivable component. In-depth examination of the gut microbiome and identification of all strains is now mainly obtained with sequencing-based techniques [127].

However, a mechanistic analysis on how specific bacterial strains may influence intestinal health has not succeeded in considering the system biology network that plays a pivotal role in disease development. The future scenarios of biomedical science will take advantage of recognizing the unique routes that lead from health to disease, according to what has been called “precision medicine”. In the pursuit of precision medicine applied to CD, a multicenter, prospective longitudinal study called CDGEMM (Celiac Disease, Genomic, Environmental, Microbiome and Metabolomic Study) is ongoing in the USA, Italy and Spain [128,129]. CDGEMM uses a multi-omic analysis approach to identify early changes in the gut microbiome of infants genetically predisposed to CD and to monitor metatranscriptomic profiles over time, correlating those profiles to other environmental factors such as mode of delivery, feeding patterns and antibiotic exposure. This study will also deeply characterize the “metabotypes” (microbe-derived metabolomes) of these “at-risk infants“ to be integrated with multi-omics profiles in the framework of system biology.

In conclusion, even though studies in both pediatric and adult patients with CD have suggested an association between altered microbiota and CD, a specific microbial signature has not been recognized. Multi-omics data from ongoing longitudinal cohort studies are eagerly awaited to further clarify this decisive field for precision medicine and primary prevention in CD.

## Figures and Tables

**Table 1:** Cross-sectional studies on Microbiota and subjects with celiac disease (CD).

Author and Reference	Journal	Year	Population	Country	Samples	Methods	Significant Findings
Sanz et al. [108]	FEMS Immunol Med Microbiol	2007	Children (*n* = 20)	Spain	Fecal samples	DGGE	The diversity of stool microbiota was significantly higher in celiac children than in healthy controls. The *Bifidobacterium* population showed a significantly higher species diversity in healthy children than in celiac patients.
Nadal et al. [109]	Journal of Medical Microbiology	2007	Children (*n* = 28)	Spain	Duodenal biopsy	Molbiol FISH	The proportions of total bacteria and Gram-negative bacteria were significantly higher in CD patients with active disease than in controls. The ratio of *Lactobacillus–Bifidobacterium* to *Bacteroides–E. Coli* was significantly reduced in celiac patients with active disease compared with controls.
Sánchez et al. [121]	BMC Gastroenterology	2008	Children (*n* = 21)	Spain	Fecal samples	VRBD agar, API20E system, PCR	Virulence-gene carriage was higher in *E. coli* isolated from CD patients compared to healthy controls.
Collado et al. [110]	J Clin Pathol	2009	Children (n 60 fecal samples and *n* = 33 biopsy samples)	Spain	Fecal samples and duodenal biopsy	qPCR	*Clostridium leptum* and *Bacteroides* were significantly more abundant in feces and biopsies of CD patients than in healthy controls. *E. coli* and *Staphylococcus* were significantly higher in stool and biopsy samples of CD patients compared to controls, *Bifidobacterium* were significantly lower in feces of CD patients as well as in biopsies compared to control children.
Ou et al. [107]	Am J Gastroenterol	2009	Children (*n* = 51)	Sweden	Duodenal biopsy	16s RNA amplification	Small intestine microbiota from CD patients did not differ from controls.
De Palma et al. [111]	BMC Microbiology	2010	Children (*n* = 44)	Spain	Fecal samples	Molbiol FISH	*Bacteroides* and *Prevotella* were higher in untreated CD patients than in controls. *Clostridium histolyticum, Clostridium lituseburense* and *Faecalibacterium prausnitzii* were higher in healthy individuals than in CD patients. *Bifidobacteria* were significantly reduced in untreated CD patients.
Schippa et al. [112]	BMC Microbiology	2010	Children (*n* = 20)	Italy	Duodenal biopsy	TGGE	A higher diversity in dominant microbiota was found in CD patients compared to controls. *Bacteroides* were significantly higher in CD compared to controls.
Nistal et al. [118]	Biochimie	2012	Adults (*n* = 21)	Spain	Fecal samples	DGGE	*Bifidobacterium bifidum* was significantly higher in untreated CD patients than healthy adults.
Sánchez et al. [122]	J Clin Pathol	2012	Children (*n* = 20)	Spain	Fecal samples	PCR ABI PRISM-3130XL Gene Analyzer	*Staphylococcus epidermidis* and *haemolyticus* were more represented in the microbiota of active CD. *Staphylococcus* spp. diversity was higher in active CD patients than in controls. The methicillin-resistant gene (mecA) was most frequently identified in *S. epidermidis* isolates from active CD than in those from controls.
Sánchez et al. [90]	Applied and Environmental Microbiology	2012	Children (*n* = 40)	Spain	Fecal samples	Schaedlr agar, 16S rRNA amplification	Active CD patients had a higher abundance of *Bacteroides fragilis* and a lower abundance of *Bacteroides ovatus* than controls.
Nistal et al. [117]	Inflamm Bowel Dis	2012	Children (*n* = 13 and Adults *n* = 15)	Spain	Duodenal biopsy	16S rRNA amplification	Bacterial richness in the upper small intestinal mucosa was higher in adults than in children.
Kalliomaki et al. [71]	JPGN	2012	Children (*n* = 19)	Finland	Duodenal biopsy	qPCR	No significant differences in the amounts or frequencies of bacteria were identified between the study groups.
Sánchez et al. [113]	Applied and Environmental Microbiology	2013	Children (*n* = 40)	Spain	Duodenal biopsy	16S rRNA amplification	Increased diversity of the cultivable mucosa-associated bacteria from CD patients compared to the diversity of bacteria from the controls. *Proteobacteria* were more abundant in active CD patients than in controls.
Cheng et al. [114]	BMC Gastroenterology	2013	Children (*n* = 20)	Finland	Duodenal biopsy	HITChip	None of the 65 genus-like bacteria was found to be significantly more or less abundant between CD versus healthy controls.
Wacklin et al. [120]	Inflamm Bowel Dis	2013	Adult (*n* = 51)	Finland	Duodenal biopsy	PCR- DGGE	Patients with CD presenting *Dermatitis Herpetiformis* shared more similar microbiota with controls than those with other clinical features of CD. Patients with GI symptoms had a higher amount of *Proteobacteria* than the patients with another manifestation of the disease or the control subjects.
de Meij et al. [115]	Scandinavian Journal of Gastroenterology	2013	Children (*n* = 42)	Netherlands	Duodenal biopsy	16S–23S ISPRO PCR	No relevant differences in small bowel mucosal microbiome composition and diversity index was found between children with untreated CD and control.
Nistal et al. [116]	Journal of Applied Microbiology	2016	Adults (*n* = 18)	Spain	Duodenal biopsy	16S rRNA gene pyrosequencing	No differences in the duodenal microbiota between untreated CD patients and non-CD controls.
D’Argenio et al. [94]	Am J Gastroenterol	2016	Adult (*n* = 35)	Italy	Duodenal biopsy	16s next generation sequencing	*Proteobacteria* were more abundant and *Firmicutes* and *Actinobacteria* less abundant in active CD than in controls. *Neisseriales* order, the *Neisseriaceae* family, and the *Neisseria genus* were significantly more abundant in active CD patients than in controls.
Bodkhe et al. [119]	Front. Microbiol	2019	Adults (*n* = 47)	India	Duodenal biopsy and fecal samples	Illumina MiSeq sequencing	In the CD group, *Helicobacter* and *Megasphaera genera* were highly abundant compared to both first-degree relatives and control group in duodenal samples. *Barnesiella* was higher in controls compared to CD and first-degree relatives in duodenal samples. *Dorea, Akkermansia* and *prevotella genera* were higher in fecal samples from controls compared to CD.

DDGE: Denaturing Gradient Gel Electrophoresis; FISH: Fluorescent in situ hybridization; VRBD: Violet Red Bile Dextrose; API: Analytical profile index; qPCR: quantitative polymerase chain reaction; TGGE: Temperature gradient gel electrophoresis; HITChip: Human Intestinal Tract Chip.

**Table 2:** Prospective studies on microbiota development in subjects at risk for CD.

Author	Journal	Year	Population	Country	Samples	Methods	Age of Sampling	Significant Findings
Sanchez et al. [124]	Applied and environmental microbiology	2011	Children (*n* = 75)	Spain	Fecal samples	DGGE	7 days, 1 month and 4 months	The *Bacteroides* diversity index was higher in formula-fed infants than in breast-fed infants, infants with high genetic risk showed a higher prevalence of *B. vulgatus*, while those with low genetic risk displayed a higher prevalence of *B. ovatus*, *B. plebeius*, and *B. uniformis*.
Sellitto et al. [125]	PLoS One	2012	Children (*n* = 34)	USA	Fecal samples	Roche/454 FLX pyrosequencing	7 and 30 days, 6 months, 8 months, 10 months, 12 months, 18 months and 24 months	Genetically at-risk for CD enrolled in this study were characterized by a low abundance of members of the *phylum Bacteroidetes*, one infant who developed CD showed high levels of lactate between 6 and 12 months of age.
Olivares et al. [69]	Microbiome	2018	Children (*n* = 20)	Spain	Fecal samples	Illumina MiSeq sequencing	4 months and 6 months	Children not developing CD showed rising bacterial diversity over time. A higher abundance of *Bifidobacterium longum* was found in control children while higher *Bifidobacterium breve* and *Enterococcus* spp. were found in those who developed CD.
Olivares et al. [79]	Gut Microbes	2018	Children (*n* = 127)	Spain	Fecal samples	16S rRNA amplification	7 days, 1 month and 4 months	A higher prevalence of enterotoxigenic E. coli (ETEC) was found in infants with the highest genetic risk compared either to those with a low or intermediate risk
Rintala et al. [126]	Scandinavian Journal of Gastroenterology	2018	Children (*n* = 27)	Finland	Fecal samples	Illumina MiSeq sequencing	9 months and 12 months	No statistically significant differences in microbiota were found between children who later developed CD and the control children without disease or associated autoantibodies.
